# Classification of microadenomas in patients with primary aldosteronism by steroid profiling

**DOI:** 10.1016/j.jsbmb.2019.01.008

**Published:** 2019-05

**Authors:** Yuhong Yang, Jacopo Burrello, Alessio Burrello, Graeme Eisenhofer, Mirko Peitzsch, Martina Tetti, Thomas Knösel, Felix Beuschlein, Jacques W.M. Lenders, Paolo Mulatero, Martin Reincke, Tracy Ann Williams

**Affiliations:** a Medizinische Klinik und Poliklinik IV, Klinikum der Universität, Ludwig-Maximilians-Universität München, Munich, Germany; b Division of Internal Medicine and Hypertension, Department of Medical Sciences, University of Turin, Turin, Italy; c Department of Electronics and Telecommunications, Polytechnic University of Turin, Turin, Italy; d Institute of Clinical Chemistry and Laboratory Medicine, University Hospital Carl Gustav Carus, Technische Universität Dresden, Dresden, Germany; e Department of Medicine III, University Hospital Carl Gustav Carus, Technische Universität Dresden, Dresden, Germany; f Institute of Pathology, Ludwig-Maximilians-Universität München, Munich, Germany; g Klinik für Endokrinologie, Diabetologie und Klinische Ernährung, Universitätsspital Zürich, Zürich, Switzerland; h Department of Medicine, Radboud University Medical Center, Nijmegen, the Netherlands

**Keywords:** 17−OH-progesterone, 17-hydroxyprogesterone, 18OH-cortisol, 18-hydroxycortisol, APA, aldosterone-producing adenoma, ARR, aldosterone-to-renin ratio, AVS, adrenal venous sampling, BAH, bilateral adrenal hyperplasia, CT, computed tomography, CYP11B1, 11β-hydroxylase, CYP11B2, aldosterone synthase, CYP17A1, 17α-hydroxylase, DHEA, dehydroepiandrosterone, DHEAS, dehydroepiandrosterone sulphate, HSD3B2, 3β-hydroxysteroid dehydrogenase type 2, H&E, hematoxylin and eosin, LC–MS/MS, liquid chromatography-tandem mass spectrometry, Macro-APA, macro-aldosterone-producing adenoma, Micro-APA, micro-aldosterone-producing adenoma, MRA, mineralocorticoid receptor antagonist, OR, odds ratio, PA, primary aldosteronism, Primary aldosteronism, Aldosterone-producing adenoma, Conn syndrome, Bilateral adrenal hyperplasia, Adrenal cortex, Steroid profiling

## Abstract

In primary aldosteronism (PA) the differentiation of unilateral aldosterone-producing adenomas (APA) from bilateral adrenal hyperplasia (BAH) is usually performed by adrenal venous sampling (AVS) and/or computed tomography (CT). CT alone often lacks the sensitivity to identify micro-APAs. Our objectives were to establish if steroid profiling could be useful for the identification of patients with micro-APAs and for the development of an online tool to differentiate micro-APAs, macro-APAs and BAH. The study included patients with PA (n = 197) from Munich (n = 124) and Torino (n = 73) and comprised 33 patients with micro-APAs, 95 with macro-APAs, and 69 with BAH. Subtype differentiation was by AVS, and micro- and macro-APAs were selected according to pathology reports. Steroid concentrations in peripheral venous plasma were measured by liquid chromatography-tandem mass spectrometry. An online tool using a random forest model was built for the classification of micro-APA, macro-APA and BAH. Micro-APA were classified with low specificity (33%) but macro-APA and BAH were correctly classified with high specificity (93%). Improved classification of micro-APAs was achieved using a diagnostic algorithm integrating steroid profiling, CT scanning and AVS procedures limited to patients with discordant steroid and CT results. This would have increased the correct classification of micro-APAs to 68% and improved the overall classification to 92%. Such an approach could be useful to select patients with CT-undetectable micro-APAs in whom AVS should be considered mandatory.

## Highlights

• Steroid profiling used for random forest modelling of micro-APA, macro-APA and BAH.
• High accuracy for macro-APA and BAH, low accuracy for micro-APA classification.
• Micro-APA classification improved using novel diagnostic algorithm.
• Potential use to select patients with micro-APA for mandatory AVS.

## 1 Introduction

Primary aldosteronism (PA) is the most frequent form of endocrine hypertension characterized by aldosterone overproduction relative to suppressed plasma renin [[1], [2], [3]]. Patients with PA have an increased risk of cardiovascular and cerebrovascular events and renal disease progression relative to patients with primary hypertension including those with similar cardiovascular risk profiles [[4], [5], [6], [7]]. This highlights the importance of early diagnosis and appropriate clinical management to minimize detrimental cardiovascular outcomes.

Unilateral PA is mainly caused by an aldosterone-producing adenoma (APA) and is potentially curable by laparoscopic unilateral adrenalectomy whereas bilateral PA (bilateral adrenal hyperplasia [BAH]) is usually treated with a mineralocorticoid receptor antagonist (MRA). These specific treatment options emphasize the central role of an accurate differentiation of APA from BAH in the diagnostic work up of PA which is usually performed by computed tomography (CT) and adrenal venous sampling (AVS) [8]. The sensitivity of CT imaging is often insufficient for the identification of APAs less than 10 mm in diameter, while AVS displays high sensitivity and specificity (95% and 100%, respectively) to distinguish unilateral from bilateral PA [9,10]. AVS is a technically-demanding and invasive procedure with non-standardized protocols and variable interpretation of results, and alternative approaches to reduce or even replace AVS for subtype differentiation in PA are currently sought [[11], [12], [13], [14], [15], [16]].

Various definitions of size of micro-APAs have been used which usually consider the CT-undetectable feature [9,[17], [18], [19], [20]]. Micro-APAs have been classified as < 10 mm in diameter, although in certain circumstances, ie, localization in an expanded adrenal limb, micro-APAs of 6 mm in diameter have been detected by CT [17]. CYP11B2 (aldosterone synthase) expression per tumour area by immunohistochemistry has been reported as higher in micro-APAs compared with macro-APAs together with a higher reported capacity for aldosterone production per tumour area [21]. CT-undetectable micro-APAs have a reported prevalence of 13%–30% in patients with APAs [9,[17], [18], [19], [20]] and therefore comprise a significant proportion of patients with APAs with potentially distinctive adrenal steroid profiles and clinical outcomes.

In the present study, we determined the liquid chromatography-tandem mass spectrometry (LC–MS/MS)-based steroid profiles of peripheral venous plasma samples from patients with micro-APAs (<10 mm in diameter), macro-APAs (≥10 mm in diameter) and BAH. Our objectives were to establish if steroid profiling could be useful to select patients with micro-APAs in whom AVS should be considered mandatory and determine if a diagnostic algorithm that integrates steroid profiling could help rationalize the use of AVS procedures in patients with PA.

## 2 Subjects and methods

The subjects included in this study were 197 patients diagnosed with PA from two referral centers (124 patients from Medizinische Klinik IV, Klinikum der Ludwig-Maximilians-Universität München, Munich, Germany and 73 patients from Division of Internal Medicine and Hypertension, University of Turin, Turin, Italy). The study was approved by the appropriate institutional ethics committees and written informed consent was obtained from all patients.

### 2.1 Diagnosis and treatment

PA was diagnosed in both Munich and Torino in accordance with the Endocrine Society Clinical Guideline using the aldosterone-to-renin ratio (ARR) as a screening test, confirmatory testing by saline infusion testing and subtype differentiation by AVS. Detailed methods for the diagnosis of PA and AVS procedures are described elsewhere [10,22]. In all patients, AVS procedures were performed under basal conditions and successful catheterization was defined with a selectivity index (adrenal vein to peripheral cortisol ratio) ≥2.0. Unilateral PA was defined by a lateralization ratio (dominant to contralateral aldosterone-to-cortisol concentration ratio) ≥4.0.

### 2.2 Classification of micro-APAs and macro-APAs and assessment of postoperative outcomes

**Fig. 1:**
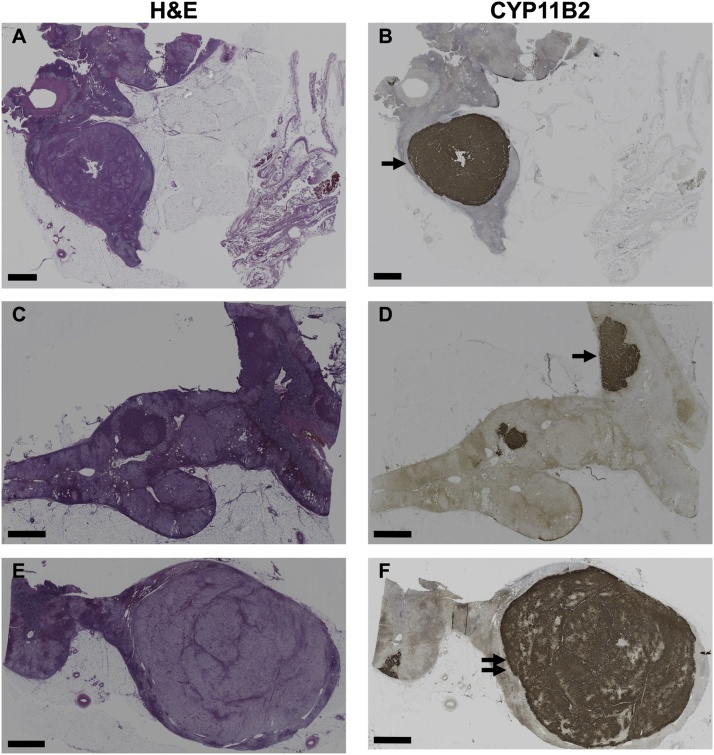
CYP11B2 immunohistochemistry micro-APA and macro-APA. The histopathology of resected adrenals from patients with a micro-APA **(Panels A, B, C and D)** or a macro-APA **(Panels E and F)** are shown with hematoxylin and eosin (H&E) staining (**Panels A, C and E**) and CYP11B2 immunostaining (**Panels B, D and F**). Micro-APAs in this study were classfied as a single micro-APA (diameter < 10 mm, as indicated by a single arrow in panel B) or the largest CYP11B2 positive nodule (with diameter < 10 mm, as indicated by a single arrow in panel D) in an adrenal with more than one nodule with CYP11B2 immunostaining. An example of a macro-APA is indicated with a double arrow in panel F. Scale bar = 2 mm. CYP11B2, aldosterone synthase; H&E, hematoxylin and eosin.

Pathology reports were used for an assessment of the diameter of the largest nodule in resected adrenals for an initial classification of adenomas as micro-APAs or macro-APAs. Macro-APAs were defined by the largest nodule diameter ≥10 mm from pathology reports alone. Micro-APAs, from the initial assessment from pathology reports, were then analysed by CYP11B2 immunohistochemistry [23] and this group included either a single microadenoma with CYP11B2 immunostaining (diameter <10 mm) or the largest nodule (with diameter <10 mm) in an adrenal with more than 1 nodule with CYP11B2 immunostaining (Fig. 1). Cases of diffuse hyperplasia were excluded. Several adrenals showed aldosterone-producing cell clusters which are defined as tight clusters of cells with strong CYP11B2 immunostaining with *zona glomerulosa* morphology located beneath the capsule and extending into the *zona fasciculata* [24,25]. The follow-up data of clinical and biochemical parameters were obtained in the surgically treated patients at 6–12 months. Outcomes were defined as complete, partial and absent clinical and biochemical success based on blood pressure measurements and antihypertensive medication dosage for clinical outcomes or plasma potassium and hormonal (aldosterone and renin) measurements for biochemical outcomes [26].

### 2.3 Genotyping and gene expression analysis

Genomic DNA was extracted from frozen adrenal tissues and DNA fragments of *KCNJ5, ATP1A1, ATP2B3 and CACNA1D* were amplified by PCR using the primers shown in Table A.1, Supplementary data and sequenced as described [27,28]. To our knowledge, PCR amplifications using these primers potentially identify all somatic APA mutations in the above genes described to date including the APA mutations described in CACNA1D listed in Prada et al. [29], with the exception of CACNA1D Glu412Asp, and the new CACNA1D mutation described recently by Nanba et al. [30].

Total RNA was extracted from adrenal tissues (tumours and adjacent cortex) and reverse transcribed as described previously [27]. Real-time PCR was performed in duplicate using TaqMan gene expression assays, and expression levels of *CYP11B2* were analyzed by 2^−ΔΔ^Ct relative quantification using *GAPDH* as the endogenous reference gene. All samples were included in the gene expression analysis with available adenoma and corresponding adjacent cortical tissue (8 micro-APAs and 32 macro-APAs). *CYP11B2* gene expression analysis indicated an absence of *CYP11B2* upregulation in 1 of the 8 samples classified as micro-APAs (tumour-to-adjacent tissue *CYP11B2* expression ratio = 0.934; genotype determined as wild type) and in 1 of 32 samples classified as macro-APAs (tumour-to-adjacent tissue *CYP11B2* expression ratio = 0.926, genotype determined as wild type). This indicates the missed dissection of the CYP11B2 positive nodule for the micro-APA and the dissection of a nonfunctional adenoma as the largest nodule for the sample classified as a macro-APA (Fig. A.1, Supplementary data).

### 2.4 Immunohistochemistry and immunohistochemical characterization

Immunohistochemistry was performed on formalin-fixed, paraffin-embedded tissue sections (3 μm) with an anti-CYP11B2 antibody (dilution 1:200, mouse monoclonal anti-human antibody, clone 17B, from Dr Celso E. Gomez-Sanchez) [23] as described previously [31].

### 2.5 LC–MS/MS-based steroid profiling

Steroid profiling of peripheral venous plasma was done by LC–MS/MS as described in full elsewhere [32] for the simultaneous measurement of 15 steroids [aldosterone, 18-oxocortisol, 18-hydroxycortisol (18OH-cortisol), 21-deoxycortisol, corticosterone, 11-deoxycorticosterone, progesterone, cortisol, cortisone, 11-deoxycortisol, 17-hydroxyprogesterone (17-OH-progesterone), pregnenolone, androstenedione, dehydroepiandrosterone (DHEA), and dehydroepiandrosterone sulphate (DHEAS)].

### 2.6 Statistical analyses

Quantitative normally distributed variables are expressed as means with SDs and quantitative non-normally distributed variables are reported as medians and interquartiles. Categorical variables are presented as absolute numbers and percentages. Comparisons between two groups were assessed by a *t* test or a Mann-Whitney test; multiple comparisons were assessed by ANOVA followed by Bonferroni test for pairwise comparisons, or Kruskal-Wallis test with pairwise comparisons. Chi-square and Fisher’s exact-tests were used to compare categorical data. A logistic regression analysis identified steroids associated with micro-APAs compared with macro-APAs and BAH with odds ratios (OR) for steroids calculated per ng/mL. An OR greater than 1 indicates an increased likelihood of micro-, macro-APAs or BAH, and an OR less than 1 indicates a decreased likelihood. IBM SPSS Statistics version 25.0 were used for statistical analyses. *P* values are given to three decimal places and are considered significant when *P* < 0.05.

### 2.7 Predictive model

Random forest algorithms were performed using MATLAB R2017b and PYTHON 2.7 to assess how concentrations of steroids in peripheral venous plasma could be used to classify micro-, macro-APAs and BAH. The prediction model can be used *via* an online tool which requires operating system Windows version 64-bit or higher and is available at https://github.com/ABurrello/Steroid-profiling-in-PA/archive/master.zip.

## 3 Results

### 3.1 Patient characteristics, outcomes and immunohistochemical characterization

Demographic and clinical characteristics at study entry and post-operative follow-up of all patients are shown in Table A.2 (Supplementary data). There was an overall difference in this cohort in the sex distribution of micro- and macro-APAs and BAH (*P* < 0.001) with micro-APAs and BAH more prevalent in men than in women (micro-APAs: 78.8% *vs* 21.2%; BAH: 73.9% *vs* 26.1%). Patients with macro-APAs displayed the highest baseline concentrations of plasma aldosterone (*P* < 0.001) and the lowest serum potassium concentrations (*P* < 0.001) relative to the micro-APA or BAH group.

Micro-APAs displayed a lower prevalence of somatic *KCNJ5* mutations (3.2% *vs* 47.4%, *P* < 0.001) and a higher proportion of the wild-type genotype (80.7% *vs* 36.2%, *P* < 0.001) compared with macro-APAs.

Complete clinical and biochemical success were less frequent in the micro-APA than in the macro-APA group (12.1% *vs* 40.0%, *P* = 0.003, 84.8% *vs* 96.8%, *P* = 0.023, respectively) whereas absent biochemical success was more prevalent in the micro-APA group (9.1% *vs* 1.1%, *P* = 0.036). In total, 8 patients (5 with micro-APAs, 3 with macro-APAs) displayed partial or absent biochemical success. CYP11B2 immunohistochemistry of these 8 resected adrenals demonstrated the presence of a solitary functional macroadenoma in 3 samples. The remaining 5 adrenals comprised 1 with a solitary micro-APA and 4 which did not have a solitary APA but showed more than one nodule with CYP11B2 immunostaining, with or without aldosterone-producing cell clusters [25], with the larger nodule considered the micro-APA.

### 3.2 LC–MS/MS-based steroid profiling

**Fig. 2:**
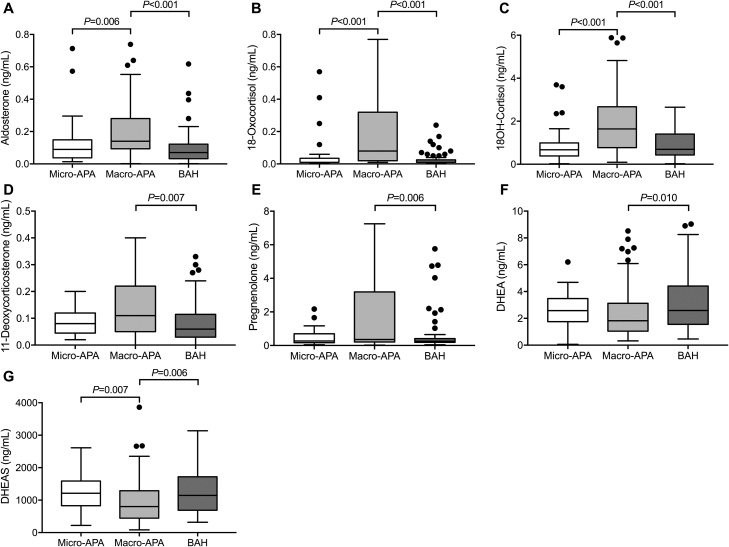
Peripheral plasma steroid concentrations in patients with micro-APAs, macro-APAs and BAH. The box and whisker plots **(Panels A–G)** represent peripheral plasma concentrations of the indicated steroids in patients with PA stratified for APA according to tumor diameter (micro-APAs ≤10 mm (n = 33) and macro-APAs >10 mm (n = 95)) and BAH (n = 69). Only steroids with significant differences in concentrations between micro-APAs, macro-APAs and BAH are shown. Horizontal lines within boxes indicate the median, boxes and whiskers represent the 25th to 77th percentiles and the minimum and maximum values, respectively, after exclusion of outliers that are defined by 1.5 times the interquartile range and are indicated by filled circles. Concentrations are indicated in ng/mL which are converted to pmol/L by dividing by the molecular weight of each steroid: aldosterone, 360.44; 18-oxocortisol, 376.45; 18-hydroxycortisol, 378.46; 11-deoxycorticosterone, 330.46; pregnenolone, 316.48; DHEA, 288.42; DHEAS, 367.50. *P* values were calculated using Kruskal-Wallis tests followed by pairwise comparisons. 18OH-cortisol, 18-hydroxycortisol; APA, aldosterone-producing adenoma; BAH, bilateral adrenal hyperplasia; DHEA, dehydroepiandrosterone; DHEAS, dehydroepiandrosterone sulfate.

LC–MS/MS-based steroid profiling of peripheral venous plasma revealed some distinct differences between patients with micro-APAs, macro-APAs and BAH (Fig. 2 and Table A.3, Supplementary data). Patients with micro-APAs had lower peripheral plasma concentrations of aldosterone (*P* = 0.006), 18-oxocortisol (*P* < 0.001) and 18-hydroxycortisol (*P* < 0.001) compared with patients with macro-APAs. The concentrations of the androgen precursor DHEAS were higher in patients with micro-APAs *versus* those with macro-APAs (*P* = 0.007). There were no significant differences in single steroid concentrations between patients with micro-APAs and BAH (Table A.3, Supplementary data). Patients with macro-APAs displayed higher concentrations of aldosterone, 18-oxocortisol, 18-hydroxycortisol (P<0.001) compared with patients with BAH (Fig. 2 and Table A.3, Supplementary data). Higher concentrations of 11-deoxycorticosterone and pregnenolone (*P* = 0.001 and *P* = 0.006, respectively) and lower concentrations of DHEA and DHEAS (*P* = 0.010 and *P* = 0.006, respectively) were measured in patients with macro-APAs relative to patients with BAH.

Potential associations of steroids with micro-APA, macro-APA and BAH were determined by logistic regression models adjusting each steroid separately for sex and age to avoid a reduction of the predictive value of variables by collinearity (Table 1). Only steroids were entered into the model which displayed a significant difference between subtype in the univariate analysis (Fig. 2, Table A.3, Supplementary data). Age had no impact on diagnosis, whereas female sex was associated with an increased likelihood of a diagnosis of a macro-APA compared with a micro-APA and with BAH.

**Table 1:** Peripheral plasma steroids associated with subtype diagnosis in primary aldosteronism.

Steroids (per ng/mL)	OR (95% CI)	*P* value	OR (95% CI)	*P* value	OR (95% CI)	*P* value
	Micro-APA *vs.* BAH (n = 101*)		Micro-APA *vs.* Macro-APA (n = 127*)		Macro-APA *vs.* BAH (n = 162*)	
Age	0.992 (0.949–1.036)	0.713	0.969 (0.928–1.011)	0.148	1.021 (0.985–1.058)	0.253
Sex (ref: female)	0.674 (0.228–1.992)	0.476	0.209 (0.076–0.572)	0.002	3.015 (1.433–6.343)	0.004
Aldosterone	11.215 (0.682–184.508)	0.091	0.422 (0.045–3.969)	0.451	877.947 (23.235–33172.977)	< 0.001
Age	0.995 (0.953–1.038)	0.813	0.966 (0.925–1.009)	0.116	1.022 (0.989–1.056)	0.201
Sex (ref: female)	0.808 (0.286–2.284)	0.687	0.197 (0.072–0.541)	0.002	3.271 (1.596–6.702)	0.001
18-Oxocortisol	1.291 (0.598–2.787)	0.515	0.761 (0.342–1.692)	0.503	2.984 (0.911–9.774)	0.071
Age	0.993 (0.951–1.037)	0.753	0.964 (0.920–1.010)	0.122	1.019 (0.985–1.055)	0.279
Sex (ref: female)	0.772 (0.275–2.171)	0.624	0.253 (0.087–0.738)	0.012	2.651 (1.241–5.662)	0.012
18-Hydroxycortisol	0.925 (0.528–1.623)	0.787	0.484 (0.289–0.812)	0.006	2.241 (1.458–3.444)	< 0.001
Age	0.991 (0.949–1.035)	0.686	0.969 (0.928–1.011)	0.141	1.019 (0.986–1.054)	0.257
Sex (ref: female)	0.694 (0.236–2.040)	0.507	0.198 (0.073–0.542)	0.002	3.225 (1.562–6.658)	0.002
11-Deoxycorticosterone	8.194 (0.300–223.625)	0.212	0.807 (0.266–2.452)	0.705	137.637 (3.052–6207.424)	0.011
Age	0.994 (0.953–1.038)	0.790	0.961 (0.920–1.004)	0.077	1.024 (0.990–1.060)	0.165
Sex (ref: female)	0.794 (0.281–2.242)	0.664	0.198 (0.071–0.554)	0.002	3.363 (1.619–6.988)	0.001
Pregnenolone	1.062 (0.799–1.413)	0.677	0.743 (0.547–1.008)	0.057	1.486 (1.149–1.923)	0.003
Age	0.978 (0.933–1.025)	0.354	0.974 (0.930–1.021)	0.274	0.988 (0.950–1.027)	0.538
Sex (ref: female)	0.786 (0.276–2.238)	0.652	0.195 (0.071–0.532)	0.001	3.651 (1.768–7.540)	< 0.001
DHEA	0.845 (0.673–1.060)	0.146	1.068 (0.868–1.315)	0.534	0.786 (0.656–0.941)	0.009
Age	0.988 (0.941–1.038)	0.641	0.983 (0.937–1.031)	0.477	0.995 (0.956–1.035)	0.793
Sex (ref: female)	0.720 (0.242–2.142)	0.555	0.245 (0.086–0.699)	0.009	2.646 (1.249–5.607)	0.011
DHEAS	1.000 (0.999–1.001)	0.658	1.001 (1.000–1.001)	0.182	0.999 (0.998–0.999)	0.048

Logistic regression analysis to identify adrenal steroids associated with micro-APAs, macro-APAs and BAH. An odds ratio greater than 1 indicates an increased likelihood of micro-APAs compared with BAH or compared with macro-APAs or macro-APAs compared with BAH, and an odds ratio less than 1 indicates a decreased likelihood. APA, aldosterone-producing adenoma; BAH, bilateral adrenal hyperplasia; DHEA, dehydroepiandrosterone; DHEAS, dehydroepiandrosterone sulphate; OR, odds ratio. There were 33 patients in the micro-APA group; 94 in the macro-APA and 68 in the BAH groups. *1 patient from the BAH group was missing pregnenolone data and was not included and 1 patient from the macro-APA group had outlier steroid profiling data.

With the adjustment for sex and age, only lower concentrations of 18-hydroxycortisol were associated with an increased likelihood of a micro-APA (OR 0.484 per ng/mL, 95% CI 0.289-0.812, *P* = 0.006). Higher concentrations of 18-hydroxycortisol were associated with an increased likelihood of a macro-APA *versus* BAH (OR 2.241 per ng/mL, 95% CI 1.458–3.444, *P* < 0.001) (Table 1). Higher concentrations of aldosterone, pregnenolone and 11-deoxycorticosterone were also associated with macro-APAs *versus* BAH (Table 1). In contrast, lower concentrations of DHEA were associated with an increased likelihood of a diagnosis of a macro-APA (OR 0.786 per ng/mL, 95% CI 0.656-0.941, *P* = 0.009) (Table 1).

### 3.3 Random forest algorithm using steroid profiling

**Fig. 3:**
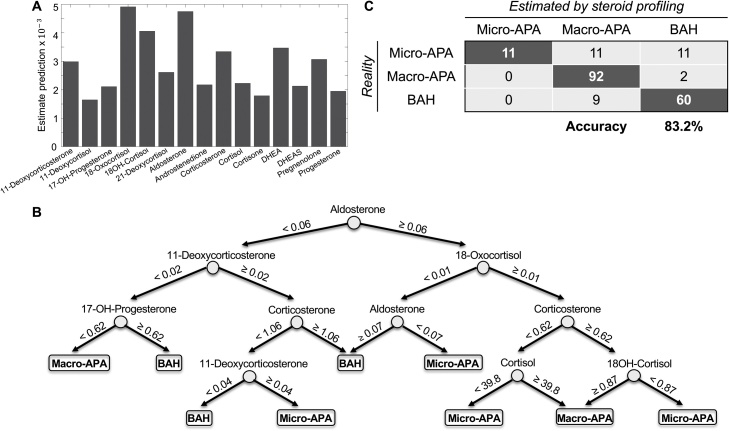
Peripheral venous steroid profiling for the classification of micro-APAs, macro-APAs and BAH. The random forest algorithm constructed performance estimates for each steroid **(Panel A)**, the first classification tree of the forest is shown for the prediction of micro-APAs, macro-APAs and BAH **(Panel B)**, and a table with the estimated classification by steroid profiling and the actual classification by AVS (to differentiate BAH from APA) and pathology reports (to differentiate micro-APAs from macro-APAs) **(Panel C)**. One patient had outlier steroid profiling results and was omitted. Numbers indicate steroid concentrations in ng/mL. To convert concentrations in ng/mL to pmol/L, concentrations should be divided by the molecular weight of each steroid. Molecular weights: 11-deoxycorticosterone, 330.46; 17-hydroxyprogesterone, 330.46; 18-hydroxycortisol, 378.46; 18-oxocortisol, 376.45; aldosterone, 360.44; corticosterone, 346.46; cortisol, 362.46. 17-OH-progesterone, 17-hydroxyprogesterone; 18OH-cortisol, 18-hydroxycortisol; APA, aldosterone-producing adenoma; BAH, bilateral adrenal hyperplasia; DHEA, dehydroepiandrosterone; DHEAS, dehydroepiandrosterone sulphate.

Random forest classification trees were used to build a prediction model for micro-APAs, macro-APAs or BAH using peripheral plasma steroid concentrations. The algorithm created 30 different classification trees to optimize the prediction model and the predictive performance of each steroid was estimated (Fig. 3A) and the first classification tree of the forest is shown (Fig. 3B). AVS and pathology reports identified a total of 33 micro-APAs, 11 of the 33 micro-APA were correctly classified by the random forest model (Fig. 3C). The correct classification of macro-APAs was 96.8% (92 of 95, with 1 patient excluded as an outlier), and 87.0% (60 of 69) of patients were correctly classified as BAH (Fig. 3C). The overall accuracy of steroid profiling for the classification of the 3 groups (micro-APAs, macro-APAs and BAH) was 83.2% (163/196), and the concordant diagnosis of APA and BAH between steroid profiling and AVS was 88.8% (174/196) (Fig. 3C).

### 3.4 Diagnostic algorithm combining steroid profiling, CT and AVS

**Fig. 4:**
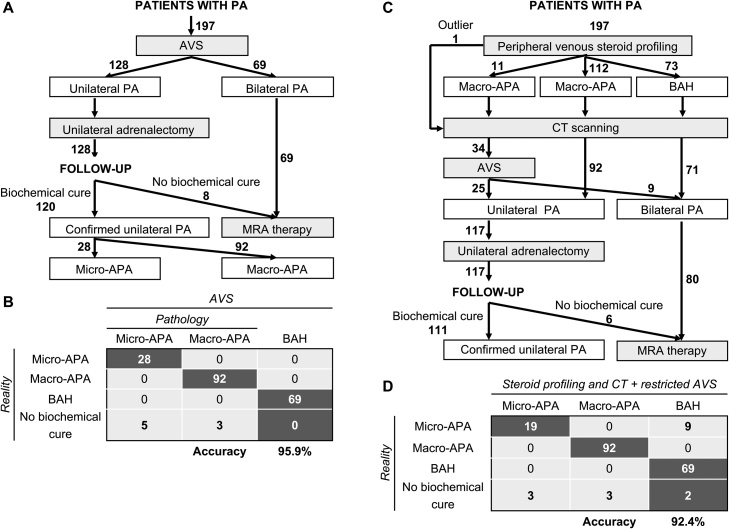
Clinical management algorithms for patients with primary aldosteronism. The AVS-based management of patients in this study is shown **(Panel A)**, and results in the accurate differentiation of APA from BAH in at best 95.9% of patients (assuming a correct diagnosis of all patients with BAH) (**Panel B**). The diagnostic accuracy of APA is assessed here by the proportion of patients with biochemical cure (120 of 128 patients with complete biochemical success). No biochemical cure was observed in 6.3% (8 of 128 patients with absent or partial biochemical success). Therapeutic management based on peripheral venous steroid profiling and CT scanning with AVS in a restricted subset of patients (patients with discordant CT and steroid profiling results) would have reduced AVS procedures by at most 82.7% (34 of 197 patients addressed to AVS) and potentially 92.4% of patients would have had an accurate differentiation of APA from BAH **(Panel C)**. The patient with outlier steroid profiling results would have been addressed to AVS. The number of patients with an absent or partial biochemical outcome would have been reduced to 6 (5.1%, 6 of 117) **(Panel D)**. Numbers in bold indicate numbers of patients. APA, aldosterone-producing adenoma; AVS, adrenal venous sampling; CT, computed tomography; MRA, mineralocorticoid receptor antagonist; PA, primary aldosteronism.

An AVS-based approach was used for the therapeutic management of the 197 patients in this study. Of these patients, 128 had laparoscopic unilateral adrenalectomy for resection of an APA and 69 were treated with MRAs for BAH (Fig. 4A). For the surgically-treated patients, 93.7% (120 of 128) displayed complete biochemical success after surgery. In patients with complete biochemical success, pathology reports indicated that the prevalence of micro-APAs was 23.3% (28 of 120 patients) and of macro-APAs 76.7% (92 of 120 patients). The remaining 6.3% (8 of 128 patients) displayed biochemical evidence of persistent hyperaldosteronism (partial or absent biochemical success). Assuming the patients with partial or absent biochemical success represent patients with a presurgical misdiagnosis of unilateral PA instead of bilateral PA, and the diagnosis of BAH with non-lateralized aldosterone secretion was accurate in all cases, the diagnostic accuracy of AVS-based management in the study cohort was 95.9% (189 of 197) (Fig. 4B).

We tested the performance of a diagnostic algorithm using peripheral venous steroid profiling and CT scanning in all patients and AVS limited to patients with discordant steroid profiling and CT scanning results (Fig. 4C). Applying this algorithm to the same cohort (n = 197 patients), peripheral venous steroid profiling would have predicted 11 patients with micro-APAs, 112 patients with macro-APAs and 73 patients with BAH. Of these patients, the 11 micro-APAs were correctly classified, 11 patients with micro-APAs were misclassified as macro-APAs, and 9 patients with BAH were incorrectly classified as macro-APAs. These 31 patients would be addressed to AVS for subtype differentiation because of potential discordant steroid profiling and CT scanning results.

An additional 2 of the 73 patients misclassified as BAH from steroid profiling instead of macro-APA would potentially be selected for AVS because of the detection of a unilateral adenoma at imaging. The patient with a macro-APA, classified as an outlier by steroid profiling, would also have been addressed to AVS. Therefore, a total of 34 patients would have AVS potentially resulting in, at the most, a theoretical reduction of AVS procedures by 82.7% (163 of 197 patients would bypass AVS assuming a normal CT morphology of the contralateral gland) with a comparable accuracy of diagnosis (92.4%) (Fig. 4D) to that of AVS-based management. The accuracy for the correct classification of micro-APAs could have reached 67.9% (19 of 28, 5 patients were excluded from the micro-APA group because they were not biochemically cured after adrenalectomy resulting in 28 patients with unilateral micro-APA), and the number of patients with incomplete biochemical cure after surgery (absent or partial biochemical success) would have decreased from 8 with the AVS algorithm (Fig. 4B) to 6 (5.1%) with the diagnostic algorithm incorporating steroid profiling (Fig. 4D). Eleven patients with micro-APAs would have been diagnosed with BAH of which 9 were micro-APAs (from patients with complete biochemical success after surgery) and the remaining 2 patients (with absent or partial biochemical success) would have received the correct treatment with a MRA (Fig. 4D).

## 4 Discussion

We measured peripheral plasma steroid profiles by LC–MS/MS in a large series of patients with PA and determined the potential utility of integrating these measurements in therapeutic management. We focussed on using this approach to identify patients with micro-APAs which are often missed by CT in those centres that rely on CT for the differentiation of APA from BAH. The similar steroid profiles of patients with micro-APAs and BAH limits the application of a method based on steroid measurements alone but when considered in a diagnostic work up that includes interpretation of CT results and AVS (restricted to patients with discordant steroid profiles and CT data), the theoretical algorithm performed almost as well as AVS alone for the diagnosis of micro-APAs.

We also analysed the post-surgical clinical and biochemical outcomes of all patients with a unilateral APA (*n* = 128) included in the study in accordance with the PASO consensus, a standarized set of criteria to define outcomes of adrenalectomy for unilateral PA [26]. A smaller proportion of patients with micro-APAs achieved complete clinical and biochemical success than patients with macro-APAs but this is likely mainly due to the sex distribution difference between patients with micro-APAs (comprising a higher proportion of males and patients with a higher BMI) and with macro-APAs [26]. The sex distribution of our cohort may partially explain the difference between our findings and those of Omura et al., who reported an increase in the proportion of patients achieving clinical cure after surgical removal of micro-APAs (*n* = 27) compared with macro-APAs (*n* = 42) [18]. Although larger nodule size was associated with complete clinical success after adrenalectomy in a multicohort study [25] and APAs with *KCNJ5* mutations, which tend to be larger than other APAs [32,33], are also associated with favourable outcomes post-surgery [34].

Patients with absent or partial biochemical success after surgery were predominantly in the micro-APA group. CYP11B2 immunohistochemistry of the resected adrenals showed that they mostly comprised more than one micronodule with positive CYP11B2 immunostaining, in agreement with the suggestion that CYP11B2 immunohistochemistry of resected adrenals may be useful as an indicator of biochemical outcomes and highlight patients which require ongoing follow-up with biochemical as well as clinical re-assessment [31]. Despite the over-representation of micro-APAs in the group of patients who were not biochemically cured after adrenalectomy, this nonetheless comprised only 15% (5 of 33) of patients with micro-APAs indicating that it is worthwhile and potentially rewarding for patients to have further work up for the identification of micro-APAs.

The small area of the resected adrenal with CYP11B2 immunostaining in micro-APAs likely explains the decreased presurgical plasma aldosterone concentrations of the corresponding patients compared with those with macro-APAs as suggested by a previous report [21]. Patients with micro-APAs also had lower plasma concentrations of the hybrid steroids, 18-oxocortisol and 18-hydroxycortisol, relative to the macro-APA group but comparable to patients with BAH. This is feasibly explained by the larger size of APA carrying *KCNJ5* mutations [35,36] and the association of *KCNJ5*-mutated APAs with an increased production of 18-oxocortisol and 18-hydroxycortisol [11]. In line with this, we show that the micro-APA group displayed a lower prevalence of *KCNJ5* mutations compared with the macro-APA group. Further, a logistic regression model with adjustment of each steroid concentration for sex and age, showed an association of higher 18-hydroxycortisol concentrations (but not 18-oxocortisol) with an increased likelihood of a diagnosis of a macro-APA compared with micro-APA and with BAH. The association of female sex with macro-APAs and with increasing plasma aldosterone concentrations is consistent with a meta-analysis report of patients with KCNJ5-mutated APAs displaying larger tumours and more pronounced hyperaldosteronism compared with patients with APAs without KCNJ5 mutations [36].

Larger APAs may have increased glucocorticoid co-secretion [37,38] which would be expected to suppress pituitary ACTH production. ACTH is the main regulator of the synthesis of DHEA and DHEAS and therefore increased glucocorticoid co-secretion from macro-APAs may partly explain the lower concentrations of these steroids observed in patients with macro-APAs compared with patients with micro-APAs and BAH. Additional studies are required to address the role of APA size and genotype on glucocorticoid co-secretion with the inclusion of plasma ACTH measurements to establish their effects on the steroid profile.

In the PASO study, 6% of 699 patients did not display complete biochemical cure after adrenalectomy for APA and this group comprises cases of PA with bilateral asymmetrical aldosterone excess with a potential misdiagnosis of unilateral PA [26]. This compares favorably with CT based decision making for adrenalectomy, where 20% of patients with APA are not biochemically cured [39,40]. Using AVS results as the reference standard, the correct diagnosis of unilateral and bilateral PA by adrenal imaging (CT or magnetic resonance) was reported as 62.2% [41]. In another study on patients with unilateral APA (diagnosed by AVS) with follow-up data, 36% of patients who were biochemically cured after adrenalectomy would have been misdiagnosed on the basis of CT results [40]. Thus, neither CT nor AVS are completely reliable and strong interest focusses on approaches to improve the performance and accessibility of subtype differentiation in PA [[12], [13], [14], [15], [16], [17]]. The potential utility of adrenal steroids in discriminating different subtypes of PA has been investigated in numerous studies with attention centering on the hybrid steroids 18-oxocortisol and 18-hydroxycortisol in urine or plasma [42]. Peripheral plasma 18-oxocortisol concentrations measured by LC—MS/MS can distinguish CT-detectable APA and BAH in patients from Japan [43]. However, in a European cohort, this method appears to be unreliable whereas a panel of 12 adrenal steroids in peripheral plasma differentiated APA from BAH with an accuracy of 80% [13]. The higher production of 18-oxocortisol in patients with *KCNJ5*-mutated APAs may explain the increased performance of 18-oxocortisol for differentiating APA from BAH in those cohorts with higher prevalences of *KCNJ5* mutations (as in Japan) compared with other populations (as in Europe) [13,43].

Genotype data in this study, as in most preceeding studies, should be interpreted with caution because recent evidence shows that when sequencing is targeted to areas of the adenoma that are positive for CYP11B2 expression, somatic mutations are detected in almost 90% of APAs [30]. Therefore, the non-targeted sequencing approach used here is a limitation of the current study with an overrepresentation of the “wild-type” genotype notably in the micro-APA group because tissue dissection may have missed the micronodule. Further, the macro-APA group was not defined by CYP11B2 immunohistochemistry but by pathology reports of the largest nodule size (which would have been the nodule sampled for sequencing) and may comprise CYP11B2-negative nodules with a wild-type genotype [33]. In these cases, a secondary nodule (or multiple APCCs) responsible for the pathologic aldosterone production [34,44] would not have been sequenced. We showed by *CYP11B2* gene expression analysis of samples classified as adenomas compared with paired adjacent adrenal cortical tissue that the above occured but in a minority of cases.

In the present study, patients with micro-APAs could not be predicted by steroid profiling alone. This was expected considering the high similarity we show of steroid profiles in patients with micro-APAs and BAH. To improve the reliability of identifying micro-APAs, we developed a hypothetical diagnostic algorithm integrating the additional consideration of CT imaging and using AVS only in patients with discordant steroid profiles and imaging data. The algorithm increased the probability of identifying micro-APAs whilst reducing the proportion of AVS procedures by up to 82.7% but achieving a comparable accuracy of diagnosis with AVS-based management.

The strengths of our study are the large patient cohort with strict inclusion criteria for screening, diagnosis and subtype differentiation from 2 expert centres that use similar AVS protocols [10,22]. All patients had peripheral venous steroid measurements by LC—MS/MS and assessment of post-surgical clinical and biochemical outcomes in surgically-treated patients using the international PASO consensus. Further strengths are the development of an accessible online tool for the prediction of microAPA, macroAPA and BAH. Limitations of our study are the non-targeted genotyping approach and the potential inclusion of nonfunctional adenomas in the macro-APA group as discussed above and the relatively small number of patients with micro-APAs compared with macro-APAs and BAH. An additional limitation is the assumption of a correct detection of macro-APAs by CT scanning in the theoretical diagnostic algorithm.

## 5 Perspectives

Steroid profiling of peripheral venous plasma could potentially be used in combination with imaging data and AVS restricted to a small proportion of patients to identify patients in whom AVS should be considered mandatory. Such an approach may be useful in centres that rely on CT for subtype differentiation in PA because such approaches can lack the required sensitivity to detect micro-APAs.

## Sources of funding

This work was supported by the European Research Council (ERC) under the European Union’s Horizon 2020 research and innovation programme (grant agreement No [694913] to M. Reincke) and by the Deutsche Forschungsgemeinschaft (DFG, German Research Foundation) Projektnummer: 314061271 - TRR 205 to G. Eisenhofer, J.W.M. Lenders, M. Peitzsch, M. Reincke and T.A. Williams and grant RE 752/20-1 to M. Reincke; the Else Kröner-Fresenius Stiftung in support of the German Conns Registry-Else-Kröner Hyperaldosteronism Registry (2013_A182 and 2015_A171 to M. Reincke) and a grant from the Ministero dell'Istruzione, dell'Università e della Ricerca (MIUR, ex-60% 2015–2016 to T.A. Williams and 2016–2017 to P. Mulatero).
