# Supplementary material for: Classification of microadenomas in patients with primary aldosteronism by steroid profiling
Source: J Steroid Biochem Mol Biol. 2019 May;189. doi: 10.1016/j.jsbmb.2019.01.008 (PMC6876277; doi:10.1016/j.jsbmb.2019.01.008)
Supplement: Supplementary file 1 [file mmc1.docx]

**ONLINE ONLY SUPPLEMENT**

**Classification of microadenomas in patients with primary aldosteronism by steroid profiling**

Yuhong Yang^1^*, Jacopo Burrello^2^*, Alessio Burrello^3^, Graeme Eisenhofer^4,5^, Mirko Peitzsch^4^,

Martina Tetti^2^, Thomas Knösel^6^, Felix Beuschlein^1,7^, Jacques WM Lenders^5,8^, Paolo Mulatero^2^,

Martin Reincke^1^, Tracy Ann Williams^1,2^

^1^Medizinische Klinik und Poliklinik IV, Klinikum der Universität, Ludwig-Maximilians-Universität München, Munich, Germany

^2^Division of Internal Medicine and Hypertension, Department of Medical Sciences, University of Turin, Turin, Italy

^3^Department of Electronics and telecommunications, Polytechnic University of Turin, Turin, Italy

^4^Institute of Clinical Chemistry and Laboratory Medicine, University Hospital Carl Gustav Carus, Technische Universität Dresden, Dresden, Germany

^5^Department of Medicine III, University Hospital Carl Gustav Carus, Technische Universität Dresden, Dresden, Germany

^6^Institute of Pathology, Ludwig-Maximilians-Universität München, Munich, Germany

^7^Klinik für Endokrinologie, Diabetologie und Klinische Ernährung, Universitätsspital Zürich, Zürich, Switzerland

^8^Department of Medicine, Radboud University Medical Center, Nijmegen, The Netherlands

* These authors contributed equally to this work

**Contents**

**Figure A.1** CYP11B2 gene expression analysis of APAs *versus* adjacent cortex

**Table A.1** Gene mutation analysis of APAs

**Table A.2** Demographic and clinical characteristics of patients with micro-APAs, macro-APAs and BAH

**Table A.3** Peripheral plasma steroid concentrations in patients with micro-APAs, macro-APAs and BAH

**References**

**Figure A.1** CYP11B2 gene expression analysis of APAs *versus* adjacent cortex


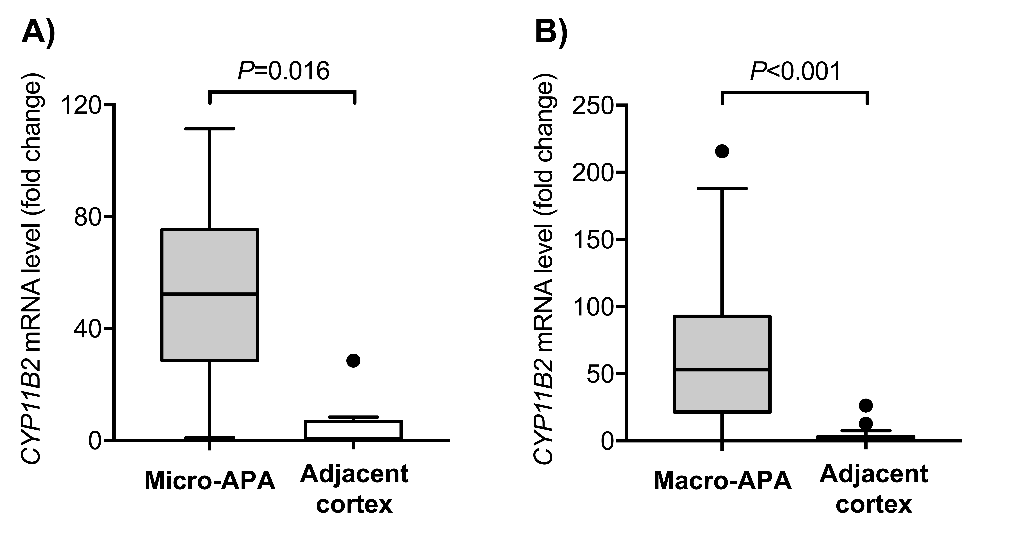


Real-time TaqMan qPCR was used to compare *CYP11B2* gene expression levels in samples classified as micro-APAs or macro-APAs compared with the respective paired adjacent cortical tissue (Adjacent cortex). Gene expression levels were calculated by the 2^-ΔΔ^Ct quantification method using GAPDH as the endogenous reference gene. We included in the analysis all samples with available adenoma and corresponding adjacent cortical tissue. The samples comprised 8 micro-APAs (1 KCNJ5 mutated, 7 wild type) and 32 macro-APAs (12 KCNJ5 mutated, 6 with ATP1A1 or ATP2B3 mutations, 2 with CACNA1D mutations and 6 wild type). The box plots show fold-changes in gene expression (mRNA levels) of CYP11B2 in the indicated tissue sample. Horizontal lines within boxes indicate the median, boxes and whiskers represent the 25th to 77^th^ percentiles and the minimum and maximum values, respectively, after exclusion of outliers that are defined by 1.5 times the interquartile range and are indicated by filled circles. Levels of significance are indicated.

*CYP11B2* gene expression analysis indicated an absence of *CYP11B2* gene upregulation in 1 of the 8 samples classified as micro-APAs (tumour-to-adjacent tissue *CYP11B2* expression ratio = 0.934; genotype determined as wild type) and in 1 of 32 samples classified as macro-APAs (tumour-to-adjacent tissue *CYP11B2* expression ratio = 0.926, genotype determined as wild type). This indicates the missed dissection of the CYP11B2 positive nodule for the micro-APA and the dissection of a nonfunctional adenoma as the largest nodule for the sample classified as a macro-APA.

**Table A.1** Gene mutation analysis of APAs

| **Gene Name** | **NCBI Reference** | **Target Exon** | **Primer sequence (5’-3’)** | **Product size** |
| --- | --- | --- | --- | --- |
| *KCNJ5* | NM_000890 | 2 | Forward: **gcttcatttggtggctcatt**  Reverse: **gagatgactgcgttgttgga** | 313 |
| *ATP1A1* | NM_000701 | 4 | Forward: tatattgccttgtaagtgctgg  Reverse: gaagtgggagacaaagacgg | 334 |
| *ATP1A1* | NM_000701 | 8 | Forward: cgtggcttccttcaggttag  Reverse: agagtgtaacattcgtgcaagc | 386 |
| *ATP2B3* | NM_021949 | 8 | Forward: ttcttccctcttcctgtccc  Reverse: ttcttaccccagtttccgag | 345 |
| *CACNA1D* | NM_001128839 | 6 | Forward: gtaaaggaggcatggttagg  Reverse: tggctcagtaaatgtgctggt | 375 |
| *CACNA1D* | NM_001128839 | 8 | Forward: **ttgaattgccctgggtgtat**  Reverse: aatgtctggcaacccctctt | 189 |
| *CACNA1D* | NM_001128839 | 14 | Forward: gtcctgcatgggtgttctga  Reverse: acgaagtgcttttcggggaa | 290 |
| *CACNA1D* | NM_001128839 | 16 | Forward: taacacttgggacggtcac  Reverse: ccatgatccacaaagcagc | 367 |
| *CACNA1D* | NM_001128839 | 23 | Forward: cacgctaactgtgcaggga  Reverse: tcagctctgcccagaagag | 279 |
| *CACNA1D* | NM_001128839 | 27 | Forward: **ccaatctacaaccaccgcgt**  Reverse: gaccaagggacagaagccaa | 198 |
| *CACNA1D* | NM_001128839 | 32 | Forward: acggttcttcctcactgtcg  Reverse: cttcagcagaggcatttggct | 338 |

The forward and reverse primers used to amplify exons 2 of *KCNJ5* and the forward primers for the amplification of exons 8 and 27 of CACNA1D anneal within the respective exons (indicated in bold) and result in partial amplification of exons. For KCNJ5, a 323 bp 5’ fragment and a 311 bp 3’ fragment of exon 2 is not amplified and for CACNA1D, exon 8, a 19 bp fragment at the 5’ end is not amplified and for exon 27, a 43 bp 5’ fragment is not amplified. The CACNA1D primers detect all mutations described in Prada et al [1] except Glu412Asp mutation that is encoded by exon 9 [2] but includes the recently described Val259Gly mutation encoded by exon 6 by Nanba et al [3].

| **Table A.2 Demographic and clinical characteristics of patients with micro-APAs, macro-APAs and BAH** | | | | | | | | | | | | | | | | | | | | | | | | | | | | | | | | | | | |  |
| --- | --- | --- | --- | --- | --- | --- | --- | --- | --- | --- | --- | --- | --- | --- | --- | --- | --- | --- | --- | --- | --- | --- | --- | --- | --- | --- | --- | --- | --- | --- | --- | --- | --- | --- | --- | --- |
| **VARIABLE** |  | |  | | | **Diagnosis** | | | | | | | | | | | | | |  | | | | | **Pairwise Comparison (*P* value)** | | | | | | | | | | | |
|  | **N** | **Total**  **(*n*=197)** | | | | **Micro-APA**  **(*n*=33)** | | | **Macro-APA**  **(*n*=95)** | | | | **BAH**  **(n=69)** | | | **Overall**  ***P* value** | | | | | **Micro-APA**  ***vs*.**  **Macro-APA** | | | | | **Micro-APA *vs*.**  **BAH** | | | | **Macro-APA**  ***vs*.**  **BAH** | | |  |  |  |  |
| Age (years) | 197 | 51 [44-58] | | | | 52 [44-57] | | | 52 [44-58] | | | | 49 [42-58] | | | 0.647 | | | | | NA | | | | | NA | | | | NA | | |  |  |  |  |
| Sex (Female) | 75 | 75 (38.1%) | | | | 7 (21.2%) | | | 50 (52.7%) | | | | 18 (26.1%) | | | <0.001 | | | | | 0.002 | | | | | 0.592 | | | | 0.001 | | |  |  |  |  |
| BMI (kg/m^2^) | 196 | 28.0±4.4 | | | | 29.3±3.8 | | | 27.1±4.6 | | | | 28.5±4.2 | | | 0.027 | | | | | 0.046 | | | | | 1.000 | | | | 0.174 | | |  |  |  |  |
| **BASELINE PARAMETERS** |  | | | | |  | | | | | |  | | | | |  | | | | |  | | | | |  | | | |  | | |  |  |  |
| Aldosterone (pmol/L) | 197 | | | 722 [466-1235] | | | 663 [516-1243] | | | 968 [583-1462] | | | | 508 [369-790] | | | | <0.001 | | | | | 0.158 | | | | | 0.086 | | <0.001 | | | |  |  |  |
| DRC (mU/L) | 124 | | | 3.5 [2.0-9.6] | | | 3.3 [2.0-9.5] | | | 3.1 [2.0-10.0] | | | | 4.1 [2.5-9.7] | | | | 0.480 | | | | | NA | | | | | NA | | NA | | | |  |  |  |
| PRA (pmol/L/min) | 73 | | | 2.6 [1.6-5.1] | | | 2.6 [2.6-6.4] | | | 2.6 [1.3-3.8] | | | | 2.6 [1.4-6.4] | | | | 0.848 | | | | | NA | | | | | NA | | NA | | | |  |  |  |
| ARR_DRC | 124 | | | 119 [55-273] | | | 166 [71-346] | | | 169 [57-357] | | | | 93 [51-146] | | | | 0.026 | | | | | 1.000 | | | | | 0.097 | | 0.051 | | | |  |  |  |
| ARR_PRA | 73 | | | 377 [204-590] | | | 366 [212-498] | | | 416 [245-810] | | | | 238 [158-460] | | | | 0.100 | | | | | NA | | | | | NA | | NA | | | |  |  |  |
| Lowest serum K^+^ (mmol/L) | 196 | | | 3.2 [2.8-3.6] | | | 3.2 [2.8-3.3] | | | 2.9 [2.5-3.2] | | | | 3.5 [3.3-3.9] | | | | <0.001 | | | | | 0.318 | | | | | <0.001 | | <0.001 | | | |  |  |  |
| Systolic BP (mmHg) | 197 | | | 158 [144-175] | | | 160 [148-175] | | | 155 [144-180] | | | | 158 [141-170] | | | | 0.510 | | | | | NA | | | | | NA | | NA | | | |  |  |  |
| Diastolic BP (mmHg) | 197 | | | 98 [89-106] | | | 100 [90-106] | | | 98 [90-106] | | | | 98 [89-105] | | | | 0.878 | | | | | NA | | | | | NA | | NA | | | |  |  |  |
| Antihypertensive medication (DDD) | 194 | | | 3.3 [2.0-5.0] | | | 3.0 [2.1-4.4] | | | 3.5 [2.0-6.0] | | | | 3.0 [1.5-5.0] | | | | 0.107 | | | | | NA | | | | | NA | | NA | | | |  |  |  |
| **CLINICAL OUTCOME** |  | | | | |  | | | | | |  | | | | |  | | | | |  | | | | |  | | |  | | | |  |  |  |
| Complete | 42 | | | 42 (32.8%) | | | 4 (12.1%) | | | 38 (40.0%) | | | | NA | | | | 0.003 | | | | | NA | | | | | NA | | NA | | | |  |  |  |
| Partial | 67 | | | 67 (52.4%) | | | 21 (63.6%) | | | 46 (48.4%) | | | | NA | | | | 0.132 | | | | | NA | | | | | NA | | NA | | | |  |  |  |
| Absent | 19 | | | 19 (14.8%) | | | 8 (24.3%) | | | 11 (11.6%) | | | | NA | | | | 0.091 | | | | | NA | | | | | NA | | NA | | | |  |  |  |
| **BIOCHEMICAL OUTCOME** |  | | | | |  | | | | | |  | | | | |  | | | | |  | | | | |  | | |  | | | |  |  |  |
| Complete | 120 | | | 120 (93.8%) | | | 28 (84.8%) | | | 92 (96.8%) | | | | NA | | | | 0.023 | | | | | NA | | | | | NA | | NA | | | |  |  |  |
| Partial | 4 | | | 4 (3.1%) | | | 2 (6.1%) | | | 2 (2.1%) | | | | NA | | | | 0.292 | | | | | NA | | | | | NA | | NA | | | |  |  |  |
| Absent | 4 | | | 4 (3.1%) | | | 3 (9.1%) | | | 1 (1.1%) | | | | NA | | | | 0.036 | | | | | NA | | | | | NA | | NA | | | |  |  |  |
| **RESECTED ADRENAL**  ***Size at pathology*** | | | | |  | | |  | | |  | | | |  | | | |  | | | | |  | | | | |  | | | | | | |  |
| Largest nodule diameter (mm) | 128 | | | 14 [9-18] | | | 7 [5-9] | | | 15 [12-20] | | | | NA | | | | <0.001 | | | | | NA | | | | | NA | | | | NA | | |  |  |
| ***Genotype*** | 125 | | | 125 | | | 31 | | | 94 | | | | NA | | | |  | | | | |  | | | | |  | | | |  | | |  |  |
| Wild-type* | 59 | | | 59 (47.2%) | | | 25 (80.7%) | | | 34 (36.2%) | | | | NA | | | | <0.001 | | | | | NA | | | | | NA | | | | NA | | |  |  |
| *KCNJ5* | 46 | | | 46 (36.8%) | | | 1 (3.2%) | | | 45 (47.9%) | | | | NA | | | | <0.001 | | | | | NA | | | | | NA | | | | NA | | |  |  |
| *CACNA1D* | 9 | | | 9 (7.2%) | | | 4 (12.9%) | | | 5 (5.3%) | | | | NA | | | | 0.310 | | | | | NA | | | | | NA | | | | NA | | |  |  |
| *ATP1A1+ATP2B3* | 11 | | | 11 (8.8%) | | | 1 (3.2%) | | | 10 (10.6%) | | | | NA | | | | 0.369 | | | | | NA | | | | | NA | | | | NA | | |  |  |
| Quantitative normally distributed variables are expressed as means with SDs and quantitative non-normally distributed variables are reported as medians and interquartiles. Categorical variables are presented as absolute numbers and percentages. *P* values are calculated using Chi-square and Fisher’s exact tests or ANOVA followed by Bonferroni tests or Kruskal-Wallis tests followed by pairwise comparisons as appropriate. APA, aldosterone-producing adenoma; ARR, aldosterone-to-renin ratio; BAH, bilateral adrenal hyperplasia; BMI, body mass index; BP, blood pressure; DDD, defined daily dose (defined daily dose is the assumed average maintenance dose per day for a drug used from its main indication in adults according to ATC/DDD Index 2018 <https://www.whocc.no/atc_ddd_index/>); DRC, direct renin concentration; K, potassium; N, number; NA, not applicable; PRA, plasma renin activity. *Wild-type indicates absence of mutations in *KCNJ5, CACNA1D, ATP1A1 and ATP2B3.* | | | | | | | | | | | | | | | | | | | | | | | | | | | | | | | | | | | |  |

| **Table A.3 Peripheral plasma steroid concentrations in patients with micro-APAs, macro-APAs and BAH** | | | | | | |  |  |
| --- | --- | --- | --- | --- | --- | --- | --- | --- |
| **Steroids (ng/mL)** | **Micro-APA**  **(n=33)** | **Macro-APA**  **(n=95)** | **BAH**  **(n=69)** | **Overall**  ***P* value** | **Pairwise Comparison (*P* value)** | | | |
|  |  |  |  |  | **Micro-APA *vs.* Macro-APA** | **Micro-APA *vs.***  **BAH** | | **Macro-APA**  ***vs.***  **BAH** |
| Aldosterone | 0.090 [0.038-0.149] | 0.140 [0.094-0.280] | 0.070 [0.033-0.123] | <0.001 | 0.006 | 1.000 | | <0.001 |
| 18-Oxocortisol | 0.010 [0.010-0.035] | 0.080 [0.020-0.320] | 0.010 [0.010-0.025] | <0.001 | <0.001 | 1.000 | | <0.001 |
| 18-OH-Cortisol | 0.670 [0.385-0.995] | 1.640 [0.770-2.670] | 0.690 [0.430-1.405] | <0.001 | <0.001 | 1.000 | | <0.001 |
| 21-Deoxycortisol | 0.079 [0.035-0.090] | 0.030 [0.010-0.091] | 0.040 [0.018-0.091] | 0.250 | NA | NA | | NA |
| Corticosterone | 2.000 [0.600-4.500] | 2.340 [1.200-4.480] | 2.470 [1.160-5.500] | 0.235 | NA | NA | | NA |
| 11-Deoxycorticosterone | 0.080 [0.045-0.120] | 0.110 [0.050-0.220] | 0.060 [0.030-0.115] | 0.002 | 0.461 | 0.637 | | 0.001 |
| Progesterone | 0.110 [0.060-0.180] | 0.130 [0.090-0.220] | 0.120 [0.080-0.215] | 0.540 | NA | NA | | NA |
| Cortisol | 115.00 [52.50-146.50] | 117.00 [66.00-152.00] | 112.00 [81.00-166.00] | 0.370 | NA | NA | | NA |
| Cortisone | 16.40 [11.95-21.45] | 16.80 [12.10-19.70] | 17.30 [13.55-21.60] | 0.566 | NA | NA | | NA |
| 11-Deoxycortisol | 0.310 [0.130-0.540] | 0.340 [0.200-0.700] | 0.290 [0.185-0.665] | 0.576 | NA | NA | | NA |
| 17-OH-Progesterone | 0.920 [0.375-1.290] | 0.710 [0.400-1.140] | 0.880 [0.500-1.295] | 0.244 | NA | NA | | NA |
| Pregnenolone | 0.270 [0.170-0.700] | 0.360 [0.210-3.190] | 0.255 [0.188-0.413] | 0.005 | 0.119 | 1.000 | | 0.006 |
| Androstenedione | 0.850 [0.590-1.160] | 0.780 [0.500-1.330] | 0.840 [0.590-1.235] | 0.825 | NA | NA | | NA |
| DHEA | 2.579 [1.751-3.481] | 1.820 [1.047-3.120] | 2.584 [1.551-4.411] | 0.009 | 0.183 | 1.000 | | 0.010 |
| DHEAS | 1210.0 [829.0-1588.0] | 800.0 [444.0-1288.0] | 1145.0 [689.5-1719.0] | 0.001 | 0.007 | 1.000 | | 0.006 |
| Quantitative normally distributed variables are expressed as means with SDs and quantitative non-normally distributed variables are reported as medians and interquartiles. Categorical variables are presented as absolute numbers and percentages. *P* values are calculated using Kruskal-Wallis tests followed by pairwise comparisons as appropriate. To convert concentrations in ng/mL to pmol/L, concentrations should be divided by the molecular weight of each steroid. Molecular weights: 11-deoxycorticosterone, 330.46; 17-hydroxyprogesterone, 330.46; 18-hydroxycortisol, 378.46; 18-oxocortisol, 376.45; aldosterone, 360.44; corticosterone, 346.46; cortisol, 362.46. 17-OH-progesterone, 17-hydroxyprogesterone; 18OH-cortisol, 18-hydroxycortisol; APA, aldosterone-producing adenoma; BAH, bilateral adrenal hyperplasia; DHEA, dehydroepiandrosterone; DHEAS, dehydroepiandrosterone sulphate; NA, not applicable. | | | | | | | | |

**References**

[1] E.T.A. Prada, J. Burrello, M. Reincke, T.A. Williams, Old and New Concepts in the Molecular Pathogenesis of Primary Aldosteronism, Hypertension 70 (2017) 875-881.

[2] Y. Yamazaki, Y. Nakamura, K. Omata, K. Ise, Y. Tezuka, Y. Ono, R. Morimoto, Y. Nozawa, C.E. Gomez-Sanchez, S.A. Tomlins, W.E. Rainey, S. Ito, F. Satoh, H. Sasano, Histopathological Classification of Cross-Sectional Image-Negative Hyperaldosteronism, J. Clin. Endocrinol. Metab. 102 (2017) 1182-1192.

[3] K. Nanba, K. Omata, T. Else, P.C.C. Beck, A.T. Nanba, A.F. Turcu, B.S. Miller, T.J. Giordano, S.A. Tomlins, W.E. Rainey, Targeted Molecular Characterization of Aldosterone-Producing Adenomas in White Americans, J. Clin. Endocrinol. Metab. 103 (2018) 3869-3876.
